# Psychosocial Challenges and Coping Strategies Among Adults Living with HIV in Ubungo Municipality, Tanzania: A Qualitative Descriptive Study

**DOI:** 10.24248/eahrj.v8i3.812

**Published:** 2025-01-30

**Authors:** Masunga K. Iseselo, Gift G. Lukumay, Idda H. Mosha

**Affiliations:** a Department of Clinical Nursing, School of Nursing, Muhimbili University of Health and Allied Sciences, Dar es Salaam, Tanzania; b Department of Community Health Nursing, School of Nursing, Muhimbili University of Health and Allied Sciences, Dar es Salaam, Tanzania; c Department of Behavioural Sciences, School of Public Health and Social Sciences, Muhimbili University of Health and Allied Sciences, Dar es Salaam, Tanzania

## Abstract

**Background:**

People living with HIV (PLWH) face numerous psychosocial challenges within the context of healthcare systems and the community where they live. This study aimed to describe psychosocial challenges and individual coping strategies among adults attending Care and Treatment Clinics (CTCs) in Ubungo Municipality, Tanzania.

**Materials and Methods:**

A qualitative descriptive study was conducted at CTCs in Ubungo Municipality, an urban setting in Dar es Salaam. Data were collected through audio-recorded in-depth interviews with 10 purposively selected participants. Audio files were transcribed verbatim and analyzed using a thematic analysis approach.

**Results:**

Difficulty in accepting HIV-positive test results, feeling desperate, fears of HIV disclosure, HIV-related stigma, and financial instability related to HIV infection were the main psychosocial challenges described by the participants. However, their main coping strategies included seeking social support, positive experiences from regular CTC attendance, adaptive coping, disregarding people’s comments, and seeking HIV-related information.

**Conclusion:**

PLWH encounter various psychosocial challenges. Feeling desperate, fear of HIV disclosure, and HIV-related stigma are the main causes of psychosocial distress among people diagnosed with HIV. Changing the individual perspectives on these challenges through effective coping strategies can improve the quality of life for PLWH. The Ministry of Health, through the National AIDS Control Program, can design interventions focused on addressing these challenges. Future research should be undertaken to quantify the magnitude of these challenges and the coping strategies in similar settings.

## BACKGROUND

In 2021, more than 38.4 million people worldwide were living with the human immunodeficiency virus (HIV), with 1.5 million newly infected.^1^ Sub-Saharan Africa has a disproportionately high HIV burden, accounting for more than 70% of the global infection.^2^ Nearly 280,000 Acquired Immunodeficiency syndrome (AIDS) related deaths have been reported in sub-Saharan Africa, and only 79% of adults were on antiretroviral therapy (ART) in 2021.^1^ Data from Tanzania show that overall HIV incidence and prevalence among adults aged 15 to 64 have declined over the past two decades. Incidence decreased from 0.64% in 2000 to 0.27% in 2017, and prevalence decreased from 7.0% in 2004 to 4.9% in 2017.^3,4^ This decrease is partly due to increased coverage of ART, with the cumulative number of people receiving ART in Tanzania increasing by 50% between 2016 and 2017.^3^ As of 2018, approximately 71% of people living with HIV (PLWH) were receiving ART.^3^ In 2014, a total of 1, 209 health facilities in Tanzania provided ART for both adults and children, 1,446,916 PLWH were enrolled in care, and 567,892 were enrolled in ART, of which about 7% were children under the age of 15.^5^

Despite advances in HIV care, PLWH face numerous psychosocial challenges after HIV diagnosis and disclosure.^6,7^ These challenges arise from the healthcare facilities and the community where the clients live. The commonly reported challenges include acceptance of HIV-positive status, disclosure concerns, ART adherence, HIV-related stigma, and/or discrimination.^8,9^ Also, the economic burden associated with access to healthcare, building an intimate relationship, mental health problems, socialisation difficulties and long waiting times at the HIV clinic are reported psychosocial challenges for PLWH.^10^ To manage this stressful situation after HIV diagnosis or disclosure, individuals may employ different coping efforts, including problem-focused strategies directed at changing a stressful situation, or emotion-focused strategies directed at changing the way one thinks or feels about a stressful situation.^11,12^ Also, they may use secrecy, denial, deception, and social withdrawal, as coping mechanisms, all of which have different implications for their well-being.^9^ Social support is the network of moral or material assistance from all facets of society, such as parents, family members, and friends.^13^ HIV coping strategies are influenced by socio-demographic, socio-cultural, and environmental aspects.^14^ In addition, coping ability may depend on health status, morality, religiosity, intelligence and individual peculiarities as well as family characteristics, social networks, economic situation, and partner relationships.^12,14,15^

Thus, in this study we aimed to bridge the insufficient understanding of the complex, multifaceted factors that affect the mental, emotional, and social well-being of individuals with HIV/AIDS. Although many studies have been conducted on the biological and medical aspects of HIV/AIDS, there has often been less focus on the psychosocial elements that influence quality of life, mental health, and overall coping mechanisms in PLWH.

Consequently, it is vital to contextualize the challenges experienced by PLWH and individualized coping strategies as reported in other studies^9,11^. This information is important for developing appropriate local interventions that address the needs of PLWH and potentially lead to better outcomes. Therefore, the purpose of this study was to describe the psychosocial challenges and the individual coping strategies among adults diagnosed/living with HIV in Dar es Salaam, Tanzania

## MATERIALS AND METHODS

### Design

A qualitative descriptive study design was used to explore and understand the psychosocial challenges PLWH face in the context of the urban setting. Due to the dynamic nature of psychological challenges among the study participants, the design was appropriate as it provided straightforward descriptions of the phenomenon.^16^ Also, we used qualitative description as it recognises the subjective nature of being HIV positive and presents the findings in a way that directly reflects or closely resembles the terminology used in the initial research question.^17^

### Setting

This study was conducted in Ubungo which is one of two newly established districts in the city of Dar es Salaam, which consists of five districts. The district was selected because of its geographical location. The city has a population of around seven (7) million and it is considered very fast-growing in Africa, and the largest commercial city in Tanzania. The first CTC program in Tanzania began in 2004. In 2018, about 6000 health facilities were registered in Tanzania to deliver HIV care and treatment services, including the provision of ART. ^18^ At the time of data collection for this study, the Ubungo municipality had a total of 37 health facilities with CTC services ^19^ of which eight (8) were purposefully selected to participate in this study.

### Participants and Recruitment Strategies

Participants were adults living with HIV, 18 years old and above, attending CTCs in the respective health facilities. The participants were purposefully selected for potential participation based on the information required in the study. Eligibility criteria were assessed after the initial contact with the research assistant (RA) at the clinic. The RA who was also experts in qualitative research facilitated the recruitment process by notifying the researchers as soon as a prospective participant completed a clinic visit. The researchers approached participants and explained the study objectives. Those who agreed to participate were asked to select a suitable interview date and time. This process considered the convenience and privacy of the participants. All the participants who were contacted during the recruitment process agreed to participate in the study.

### Data Collection Procedure

The data were collected between May and July 2020 using the in-depth interview (IDI) method. A semi-structured interview guide was used during data collection. The in-depth interview guide was developed by the first author through a literature review and was shared among all authors for the improvement of the content. All authors who were experts in mental health, and social science and had experience in qualitative research conductedthe interviews. The interviews were audio-recorded after obtaining consent from the participants. The interview took place at the health facility in a special room that was set for this purpose. The data collection activities took place in a well-lit room to enable the interviewer to capture the nonverbal cues from the participants. In addition, the room was free from external voices.

Before the commencement of data collection, we pretested the interview guide to determine if the questions were clear, understandable, and could be answered by the participants. The interviewers used interviewing skills such as outlined by Britten.^20^ Such skills increased cooperation and a sense of acceptance among the participants. The data collection continued until information saturation was attained as proposed by Hennink and Kaiser.^21^ This was when no new information was obtained after adding new participants. Data collection ended after the 10^th^ participant. Each interview lasted for an average of 60 minutes. Demographic information for each participant was collected after the interview.

### Data Analysis

Before analysis, the audio-recorded data were transcribed verbatim by RA. The authors cross-checked the transcription process by listening to the original audio and comparing them to the transcripts to ensure the exercise was correctly performed. Some typographic errors and omissions which were identified were corrected. NVivo 12 Software (QRS International) was initially used to facilitate analyses. Thematic analysis was used to analyse the data as proposed by Braun and Clarke.^22^ Transcripts were read by all research team members to identify major themes and to inform the development of a coding scheme to categorize the data. The final coding scheme included both a priori themes and those which emerged from preliminary readings of the transcripts. The codebook guided the coding process, which was completed by two members of the research team (MKI and GGL). The two coders compared coding for four interviews to ensure coding reliability and to verify understanding of the codebook^23^ and then coded the remaining interviews independently. After coding all interviews, the research team further discussed the emergent themes in the context of coding. Themes relating to psychosocial challenges and coping strategies that emerged from the data were explored through an iterative process using techniques described by Miles and Huberman.^24^ Data were further organized into themes and sub-themes. Data reduction methods were employed to extract the overarching narrative from the most pertinent data supported by participant’s quotes.

### Ethical Approval

Ethical approval to conduct this study was obtained from the Institutional Review Board (IRB) of Muhimbili University of Health and Allied Sciences (MUHAS) with Ref. No. MUHAS-REC-2-2020-093. Further, permission to conduct the study was obtained from the Ubungo Municipality Council. Before the commencement of the study, all participants were informed about the objectives, expected benefits, risks, and significance of the study. Participants were also informed about their right to refuse participation in the study at any time and were assured of the confidentiality of the collected information. Upon the participants’ agreement to participate in the study, written consent was obtained from the participants before undertaking data collection. Approval for audio recording was also obtained before starting the interviews.

## RESULTS

### Socio-demographic Characteristics of Study Participants

A total number of 10 people living with HIV were interviewed in Ubungo municipality. Of these, six (6) were females. In this study, eight (8) participants were married. The mean age of the participants was 38 and the standard deviation (SD) was 8.37 (Table 1).

**Table 1:** Social-demographic Characteristics of Study Participants

SN	Participants	Marital status	Sex	Age
1.	P01	Married	Female	42
2.	P02	Married	Female	38
3.	P03	Married	Male	42
4.	P04	Married	Male	42
5.	P05	Married	Male	27
6.	P06	Married	Male	52
7.	P07	Single	Female	39
8.	P08	Married	Female	37
9.	P09	Single	Female	20
10.	P10	Married	Female	41

### Themes Identified

Difficulties in accepting HIV-positive test results, feeling desperate, fears of HIV disclosure, HIV-related stigma and financial instability related to HIV infection were the main psychosocial challenges described by the participants. However, their main coping strategies included seeking social support, positive experiences from regular CTC attendance, adaptive coping, disregarding people’s comments, and seeking HIV-related information.

### Topic 1: Psychosocial challenges in living with HIV

#### Difficulties in Accepting HIV positive results

Participants expressed fear of their new HIV serostatus that they disputed with some of the health care providers. They reported having argued with the nurses and doctors that they did not want to initiate ARV treatment as recommended in the national HIV management guideline. Some of them disappeared completely and did not return to the clinic for follow-up and counselling for possible initiation of ARV medication as evidenced by the following participants:

*“That is since I found out… I had great fear, first I was so afraid that I was even prescribed medicine, I argued with them* [health care providers] *badly. I told them to allow me not to start the medication* [ART]. *However, they continued calling me regularly because they had taken my mobile phone number”* (P04, a married man, aged 42).

Some participants expressed fear of being sick every time and that their lives would have ended there. Also, they mentioned several challenges that they might have encountered when diagnosed with HIV infection including fear of being rejected by their sexual partners as in the statement below:

*“There are many challenges because there are times when you may feel very uneasy and say, I am truly HIV positive! For that fear may come that, now, if my husband knows it now I will be divorced”* (P02, a married woman, aged 38).

#### Feeling Desperate

When the participants received HIV test results, they had different psychological responses to the positive results. The participants reported being confused, crying and losing direction towards their own lives when diagnosed with HIV. Some stated they were overwhelmed by the situation to the extent that they lost their self-esteem and that the situation was so hard for them to live as described below:

*“I cried a lot. Because when I was diagnosed with HIV, at first, I was confused. I did not even want to eat. It reached a point where I thought of killing myself, you see. Because I was no longer of any use in the world”* (P02, a married woman, aged 38)

Participants reported feeling abandoned by their sexual partners when they recognized that they were HIV positive. Also, the presence of children and other dependents was stated to cause a lot of psychological distress among HIV sero-reactive individuals. They expressed disbelief at what had happened to their entire life as many challenges caused them to feel uneasy. This feeling was accompanied by frequent thoughts of being left by their spouses as expressed by the following participant:

*“But what was discouraging, especially when I looked at my partner, was that he would not be able to live with me anymore. I thought about my children, who will take care of them if I die?”* (P02, a married woman, aged 38).

#### Fear of HIV Disclosure

Most participants reported that they might not get married if their HIV serostatus were disclosed. They were afraid of telling others because they felt ashamed, worried about being rejected, or feared that people they told would not keep the information confidential. They described it as not always easy to talk about having HIV. They added that, when they were diagnosed with HIV they felt that they would die at any time. For example, one participant had this to share:

*“Another one finds that he is sick, stressed and thinking that now I have a problem so that my life has ended there, that at any moment I will die”* (P08, a married woman, aged 37).

However, some participants expressed different feelings about disclosure. They stated that they would prefer to disclose their HIV status themselves rather than anyone else advertising it in public without their consent. They said that it is more painful to see people whom they trusted talking about their HIV status with other people. They added that HIV self-disclosure was less painful as it increases confidence and may portray neutrality of the information as stated below:

*“…because it is better to explain it to yourself, I take this medicine and life goes on. But when someone starts advertising your health status without your consent, it hurts. Because people’s eyes, people’s fingers were giving me trouble. You feel as if the whole world knows*” (P08, a married woman, aged 37).

#### HIV-related Stigma

Participants described that people around them had negative attitudes towards them after recognizing that they were HIV positive. They revealed that getting HIV was a prejudice that came with labelling, as they were part of a group that was believed to be socially unacceptable. They added that stigma negatively affected their interaction with other people. One participant stated:

*“Although in the family I am not the first to get HIV, this stigmatizing behaviour created an image as if I had searched for it (HIV). This is because when he* (brother-in-law) *drinks alcohol he decides to tell people about my HIV status in public”* (P02, a married woman, aged 38).

However, some participants narrated that HIV-related stigma had decreased considerably compared to the beginning of the HIV infection. They verbalized that the stigma was high such that they were not involved in some social activities such as eating together or carrying one’s child or babies. People’s reaction was contributed by the perception that HIV can be infected by touching and sharing utensils. One participant had this to say:

*“Previously, life was very tough if you become known to be HIV positive. Because even if you touch a child, you are told that, you will infect him with HIV. This affected us a lot”* (P08, a married woman, aged 37).

It was further reported that many participants avoided meeting with people in the community or social gatherings due to fear of prejudices or negative attitudes about HIV. They had experienced different occasions where they could be insulted, rejected, or gossiped about and excluded from social activities. For example, one participant opined this:

*“There was a day when we were sitting at the bus stand, and someone told me, I cannot talk to you, first, you are HIV positive. I said, yes! But I asked him, are you sure if I am HIV positive? I should take you to the court”* (P04, a married man, aged 42).

#### Financial Instability Related to HIV Infection

Participants reported facing financial problems after recognizing that they fall sick and recognize that they were HIV positive. This was particularly prominent for those who had petty business as a mere source of income. They stated that, economically, all businesses had to collapse including that of the partners. One participant who was a food vendor commented:

*“Now that thing* [business capital] *is gone, during that period I was sick until I started to get up* [getting well]. *Because I was not allowed to start engaging in the business activities or moving around to sell food; considering that my businesses were related to cooking”* (P10, married woman, aged 41).

Some participants described having stayed at home for a long time due to other co-infections. They said that co-infection needed longer and more intensive treatments. One participant who was diagnosed with tuberculosis (TB) as a co-infection reported having stopped her business until she had stabilized after taking proper treatments. One participant lamented:

*“I was very stuck because I was working but I had to stay at home first until I started taking the medicine* [Anti-TB drugs]. *I resumed my business when I got used to the medicine”* (P07, single woman, aged 39).

Participants also accounted that the source of the financial problem was due to body weakness because of HIV infection. They described that, once they went to work after HIV diagnosis, they felt weak, feebly unresponsive, and practically unmotivated to work. They mentioned the cause of such a situation could be either the HIV infection or the ART medication they were taking. This was described by one participant:

*“In the beginning, when I go to work, I feel weak, I could not work. I was not interested in work and was a satisfied person. There was no motivation for work and every time I wanted to work, I felt lazy”* (P04, a married man, aged 42).

### Topic 2: Coping Strategies for people living with HIV

#### Seeking social support

Participants reported that social support played a key role in lessening psychological problems, general HIV care, and helping participants regain hope. Participants described the main source of support were the sexual partners, parents and healthcare providers as described in their respective sections.

#### Support from sexual partners

Some participants expressed partner support as a huge source of comfort after HIV diagnosis. They verbalized that having been diagnosed with HIV was a problem that consumed most of their time thinking of future life. Also, they added that social support from parents was mentioned as a support system received by the participants when they succumbed to distressing situations such as HIV diagnosis.

*“But later my husband became the main supporter and pleased me a lot. He counselled me about the emotional problems I was having, thus preventing me from making untoward decisions. …. he was encouraging me until I was comfortable”* (P02, a married woman, aged 38).

The participants verbalised that, support from their partners was relieving as it helped them with psychological distress. So, described that partners encouraged them to accept their HIV status which could help to reduce the distress. The participants also emphasized that the partners’ role was paramount in comforting the spouse about the diagnosed illness. One participant commented:

*“But later my husband the biggest comforter. He informed me about other people including our relatives who were diagnosed with HIV but now are doing fine. He insisted on admitting that I had an HIV infection. This helped me to move on”* (P07, married woman, aged 39).

In addition to relieving distress, the partners acted as middlemen between the healthcare professionals and the person with HIV. The constant communication with the CTC staff helped the participants gain new insights and techniques for handling distressing situations of HIV infection. One participant shared the following:

*“I think he* [husband] *was getting advice from him* [the doctor], *and he was coming to work on it for me. That is how it went until my condition improved. Because I was a person to be assisted for everything, and my condition was very bad because I was diagnosed with HIV infection in a late stage of AIDS” (*P02, a married woman, aged 38).

#### Support from Healthcare Providers

The participants stated that the healthcare providers used to remind them of the clinic days particularly when is due. They said that sometimes they could forget to attend the clinic, because of the healthcare providers’ commitment and regard, they took responsibility for calling them through their numbers that were in the clinic record. They emphasized that being a good compliant made them feel that they are human and they are respected as other people which makes them comply with the medication schedule.

*“The nurse that we met at the clinic always likes to remind us on our clinic day. You see, tomorrow is your date so he will look for you today because we are human beings; you can forget yourself, then he spends his money to call you, that you know that tomorrow is your clinic date, that’s it*.” (P05, a married man, aged 27).

Also, participants reported that the support provided by healthcare providers was a pillar to the improvement of their health status. Some participants who were hopeless in their future lives witnessed that the healthcare providers tried as much as possible to counsel them to regain their hope. The healthcare providers seemed overwhelmed; they used to call their partners for additional support and information for the clients. They added that the healthcare providers explained to the partners all the needed information about the client’s condition. One participant had this to say:

*“But it was time until these sisters* [health care providers] *have done everything to comfort me. That is until they decided to call my wife, they sat down with her… and explained to her that there is one, two, three. But I had completely decided it should be like that* [die]*”* (P04, a married man, aged 42).

#### Support from Parents

The role of parents could not be underestimated. One participant emphasized the support provided by his mother in her future life. The participant expressed feeling guilty and worthless in the eyes of people because of HIV infection. For example, one participant said this:

*“I was ready to do anything just to die because I was saying how will I live with people? But my mother told me, no. Some people just live so long with HIV that they do not die. My mother loved me and wanted me to live. She continued to comfort me wherever I was, she was with me”* (P09, single woman, aged 20).

#### Positive Experiences from Regular CTC Attendance

Regular CTC attendance was also a learning process through interacting with other PLWH. They observed that some of their colleagues had poorer conditions than the participants which made them realize that they did not need to complain about the existence of the virus in their bodies as in the statement below given by one participant:

*“The more I came to the hospital, the more I knew that my condition was better than others. Another person tells you that my husband does not have the infection… I have this problem. Or someone tells you that I have come to take medicine for my husband, my husband has a problem (HIV), but I do not have it* [HIV]. *It has built me the ability to discover that living with HIV infection is not the end of life. You just go on with your life as usual, like other human beings”* (P02, a married woman, aged 38).

Another participant added on the fact that he is used to being HIV positive because there are others people who are positive at CTC. He said:

*“Right now I am getting used to it. When you see each one of us here* [in the CTC clinic], *many of us are HIV positive, so I am comforted”.* (P04, a married man, aged 42).

#### Using adaptive coping strategies

Participants asserted that critical thinking about HIV and the fact that there is a way of improving their health despite the virus being in their bodies was one of the adaptive coping strategies that improved their coping strategies. This empowered them with the ability to think logically and avoid the situation of living with HIV-related stress. Additionally, participants stated that commitment to follow instructions and acceptance of the HIV positive status was mentioned as important for adaptive coping strategy. They asserted that some people with HIV infection lived longer after accepting their condition and adhering to professional advice and counselling. They emphasized that living by following individual values was more important than waiting to be accepted by other people as shown below:

*“…others have many years of living with these problems* [HIV], *and when you are informed that you are HIV positive, at first you are surprised. If a person does not die, it means that you have to accept yourself, that this is my life”* (P08, a married woman, aged 37).

Participants compared HIV infection with other chronic conditions such as cancer, diabetes and hypertension. This is the highest way of adaptive thinking that participants expressed to help them cope with a debilitating disease. Furthermore, they said, it is possible to live without anything of value. However, they described that living with other chronic conditions such as cancer and diabetes may not be the same as that for the person living with HIV as described below:

*“…it is better to get infected with HIV than that get cancer, hypertension or diabetes. Even in my activities, I try to advise them that whatever you meet in front of you, is for you. It should not change you”* (P06, married man, aged 52).

#### Disregarding People’s Comments

Participants also described that ignoring people’s comments and derogatory words was the central part of the coping strategy for PLWH. They stated that when you recognize that someone is belittling you because of your HIV status, it is better not to pay attention to him/her. One participant had this to share:

*“But you realize that I am like this, just leave them and accept your situation… when someone insults me, I don’t even talk to him… I just keep silent. Because even if you argue with him, you will not gain anything. So you just keep quiet. And just face it by ignoring it”* (P09, single woman, aged 20).

#### HIV-Related Information-Seeking Behaviour

The participants also verbalized that, the frequent education provided in the CTCs helped them to cope with living with HIV. They said the education and information gained from healthcare providers made them learn that AIDS does not kill. They argued that opportunistic infection is the condition that contributes to the death of a PLWH as commented by the following participant:

*“I have learned a lot from these healthcare providers. HIV does not kill. What kills are opportunistic infections because if you stop taking medicine, you will get opportunistic diseases. At the end of the day, you may lose your life.* ” (P02, a married woman, aged 38).

The participants expressed that, information is “power”; meaning that, if they were well informed, they would have understood the reason behind for services they received from the CTC clinic. Although some participants got opportunities to listen to the news on HIV from different media, they said that it was not enough for them to understand and clear all the distress related to HIV infection. Furthermore, most of them do not get time to follow the news on different platforms as stated below:

*“Now some* (PLWH) *have education and listen to the media, and they follow things like that. However, my partner and I are always busy. We do not have time to listen to the media. But here* [at CTC], *you may be surprised that just as people are forced to wear masks during Covid 19 outbreak, they are also forced to take medicine.”* (P08, a married woman, aged 37).

Participants also stated that ARTs-related education provided to the participants is part of improving coping strategies. They suggested the education provided by healthcare providers be improved. They said that non-compliance to ART might be caused by little understanding of the importance of HIV medications among clients. To enhance well-being, healthcare providers should have at least 30–40 minutes to provide ART-related education every time the client comes to the clinic for a medication refill. One participant said this:

*“… maybe the education should be improved because you might find clients who initially had agreed to take ART medicine, but they do not know about the importance of taking the medicine. … at the end of the day, when they reach there* [at home], *they just throw them* [medicine] *away… when they come back, the number of viruses* [viral load] *has gone up causing more health and psychological problems.”* … *they should be given education maybe within 30 or 40 minutes* …” (P04, a married man, aged 42).

## DISCUSSION

This study aimed to describe the psychosocial challenges and the individual coping strategies among adults diagnosed/living with HIV. Challenges of accepting HIV-positive test results, feeling desperate, fears of HIV disclosure and HIV-related stigma were the main psychosocial challenges described by the participants. Seeking social support, positive experiences from regular CTC attendance, adaptive coping and using HIV-related education and information were the coping strategies for people living with HIV reported in this study.

Regarding the challenges of accepting HIV positive test results as reported in this study indicates that receiving an HIV diagnosis is a life-threatening process with many emotions; sadness, hopelessness, or anger. This is because, acceptance of HIV seropositivity and engagement in HIV care is a complex process involving intrapersonal, interpersonal and environmental influences ^25^. The study participants’ initial emotions were quite traumatic and demoralizing. The findings in our study are comparable to those reported by Owusu in Ghana ^26^ in that both had negative psychosocial reactions, including thoughts of committing suicide. Additionally, according to our findings, being HIV positive can put one at risk for social rejection and divorce. This finding is similar to that reported in other studies^8,9,26^ whereby acceptance of HIV-positive status and disclosure problems were the main concern. However, nearly all of the participants in this study started and continued their HIV/AIDS healthcare after experiencing the new diagnosis and settling down. This supports earlier claims that such early reactions to newly diagnosed HIV-positive diagnosis generally fade away over time.^25,27^

The financial problems experienced by the participants in this study indicate that HIV/AIDS is a threat to social and economic development. Familial or individual financial problems might arise because of weakening social and familial support networks, decreased engagement in productive activities as a result of HIV/AIDS in the family, depleted family income owing to loss of job, and inadequate illness management.^28,29^ This has great implications for HIV care and the well-being of PLWH in low resource-setting countries like Tanzania. The economic problems for PLWH in this study are comparable to those reported in India,^29^ Vietnam^30^ and Malaysia.^31^ However, the financial problems in Vietnam may be complicated by out-of-pocket payments for HIV/AIDS services, unlike our study settings in which free services are offered to PLWH. Participants in the current study revealed the cost of transport, inadequate time for productive activities, and cost of purchasing other drugs for opportunistic infections which are not covered in the free packages of CTC services were the main challenges. The provision of social support and income-generating programmes to HIV-affected individuals and their families is a viable solution to financial problems in the study population. However, this needs further in-depth exploration to come up with a solid conclusion.

The findings from this study revealed HIV-related stigma among the study population. The stigma of HIV and AIDS is one of the social processes that adversely affects multiple facets of engagement in HIV-related care as well as HIV serostatus disclosure and social support.^32–34^ According to a psychological inhibition model,^35^ PLWH believes they are unable to report their seropositivity to others for fear of the possibly unpleasant repercussions of disclosing their discredited status. This model can help explain the negative impacts of stigma reported in the current study. Also, the misconception that HIV can be transmitted through sharing personal and household cutlery indicates that the people in the study setting have a poor understanding of the methods of HIV transmission. Myths and misconceptions exacerbate stigma and discrimination among PLWH in Tanzania. Similar findings had been reported in other African countries including Ghana,^36^ Nigeria^37^ and Malawi^38^ whereby myths and misconceptions about HIV transmission were associated with a low level of education of the study participants. This indicates that education is required to dispel myths and misconceptions related to HIV infection. The HIV-related stigma needs to be reduced or eliminated to increase participation in health-promoting activities among PLWH including attending CTCs. Different measures should be imposed to reduce HIV-related stigma. Dunbar et al ^39^ reported that self-acceptance, motivational activation for behaviour change, socialization and empowerment were the main approach to stigma reduction among PLWH. Our study suggests a need for well-designed intervention studies that are tailored to people living with HIV at CTCs for stigma reduction.

Concerning the coping strategies for PLWH, seeking social support was one of the methods used by the study participants to revert to the psychological state after testing HIV positive. Spouse was the main source of support for their partners indicating that HIV infection has a significant effect on the couple’s romantic relationship. In their study, Remien et al ^40^ demonstrated that HIV care and treatment retention were improved by a couples-based strategy that focused on the primary love relationship. In our study, the partners acted as middle-person between the healthcare professionals and the person living with HIV; thus, equipping them with strategies and techniques for HIV care. As reported by Hatteberg, ^41^ social support has a direct effect on health and an indirect effect on buffering stress caused by being HIV positive. Also, our findings revealed that family members particularly the parents had a significant contribution in comforting their relative on the effects of HIV diagnosis. It is documented that family support plays an important role in the lives of PLWH because they support direct care.^42–44^ Unlike the findings reported in Zimbabwe,^45^ whereby increased parental attentiveness to the needs of their children is the main driver for engagement with HIV services, our study demonstrates that by getting support from parents, PLWH feel more valued and can face the problems that exist in the community.

Adaptive coping strategies used by participants in this study indicate that coping is a self-regulatory effort aimed at reducing the adverse consequences of stress caused by HIV infection. Efforts to engage with the source of stress enhance a sense of personal control over the situation and adapt to the situation have been similarly reported in other studies.^46–49^ Although a coping strategy by disregarding people’s comments as revealed in our study increased levels of life satisfaction among PLWH, it is reported to be associated with negative psychological outcomes such as depressive symptoms, anxiety, emotional distress, and a less positive state of mind.^49^

In our study, HIV-related information-seeking behaviour can be described by the participants’ expectation of improved quality of life after receiving the right information. The enhanced quality of life as a result of using ART as well as a source of coping mechanism among PLWH has been reported in other studies elsewhere.^50,51^ Also, the role ARVs-related education as part of coping strategies in our study calls the healthcare providers to increase contact time with the client during clinic visits. These findings and the participants’ suggestions have broader implications for other HIV care in Tanzanian clinics. Thus, in-depth explanations about HIV, and its treatment, should be the main focus of this counseling to improve coping with HIV infection among the study population. Contrary to our findings, a study in Kilimanjaro, Tanzania reported the use of alcohol, unavailability of food, stigma, and disclosure concerns as the main barriers to effective use of ART^52^ hence affecting coping with HIV. The participants’ suggestion that HIV-related information be provided at the CTCs shows that the PLWH need more knowledge of how to handle the stress caused by HIV diagnosis.

Generally, the study findings revealed that PLWH encounter various psychosocial challenges which might impact their ability to adhere on ART and their health in general. Interventions should therefore focus on addressing these challenges.

### Limitation

The conclusions of this study may not necessarily apply to other places outside of the study’s specific geographic area in Tanzania. The study’s participants were adults getting HIV care from CTCs, therefore the opinions they shared may not reflect those of all adults with HIV in the study area as a whole. Moreover, socially desirable answers may have occurred due to face-to-face interviews during data collection. Finally, since we were interested in the psychosocial challenges and coping strategies of adults living with HIV themselves, we only used a single qualitative data collection method rather than multiple data sources which would have allowed for the triangulation of the information provided.

## CONCLUSION

PLWH encounter various psychosocial challenges. Feeling desperate, fear of HIV disclosure and HIV related stigma are the main cause of psychosocial distress among people diagnosed with HIV. Changing the individual perspectives on these challenges through effective coping strategies can improve the quality of life for PLWH. The Ministry of Health, through the National AIDS Control Program can design interventions focused on addressing these challenges. Future quantitative research should be undertaken to quantify the magnitude of these challenges and the coping strategies in similar settings.
